# Obesity and Breast Cancer: Interaction or Interference with the Response to Therapy?

**DOI:** 10.3390/curroncol30010094

**Published:** 2023-01-16

**Authors:** Silvia Riondino, Vincenzo Formica, Elena Valenzi, Cristina Morelli, Valeria Flaminio, Ilaria Portarena, Francesco Torino, Mario Roselli

**Affiliations:** 1 Department of Systems Medicine, University of Rome Tor Vergata, Via Montpellier, 1, 00152 Rome, Italy; 2 Medical Oncology Unit, University Hospital Tor Vergata, Viale Oxford, 81, 00133 Rome, Italy

**Keywords:** breast cancer, obesity, BMI, aromatase inhibitors

## Abstract

Background: Aromatase inhibitors (AI) are widely used for treating hormone-sensitive breast cancer (BC). Obesity, however, due to aromatase-mediated androgen conversion into estradiol in the peripheral adipose tissue, might impair AI inhibitory capacity. We aimed at identifying a cut-off of body mass index (BMI) with significant prognostic impact, in a cohort of stage I-II BC patients on systemic adjuvant therapy with AI. Methods: we retrospectively evaluated routinely collected baseline parameters. The optimal BMI cut-off affecting disease-free survival (DFS) in AI-treated BC patients was identified through maximally selected rank statistics; non-linear association between BMI and DFS in the AI cohort was assessed by hazard-ratio-smoothed curve analysis using BMI as continuous variable. The impact of the BMI cut-off on survival outcomes was estimated through Kaplan–Meier plots, with log-rank test and hazard ratio estimation comparing patient subgroups. Results: A total of 319 BC patients under adjuvant endocrine therapy and/or adjuvant chemotherapy were included. Curve-fitting analysis showed that for a BMI cut-off >29 in AI-treated BC patients (*n* = 172), DFS was increasingly deteriorating and that the impact of BMI on 2-year DFS identified a cut-off specific only for the cohort of postmenopausal BC patients under adjuvant therapy with AI. Conclusion: in radically resected hormone-sensitive BC patients undergoing neoadjuvant or adjuvant chemotherapy and treated with AI, obesity represents a risk factor for recurrence, with a significantly reduced 2-year DFS.

## 1. Introduction

Luminal-like breast cancer (BC), characterized by hormone-receptor (HR) positivity [1], represents the most frequent molecular subtype diagnosed in women of all ages [2]. In early stages (I and II) BC, breast-conserving therapy (BCT) consisting of lumpectomy or quadrantectomy followed by postoperative radiotherapy by means of whole breast irradiation has proven superior in terms of both disease-free survival (DFS) and overall survival (OS) as compared to total mastectomy [3]. Patients with hormone-responsive tumors, characterized by the expression of estrogen or progesterone receptors in at least 1% of cells, are candidates for adjuvant endocrine therapy, although the 1% threshold has recently been questioned [4]. The choice of endocrine therapy in patients with radically resected BC is based on the assessment of their menopausal status [5]. In premenopausal women, the drug of choice was Tamoxifen for at least 5 years, with associated ovary function suppression (OFS) [6], while latest evidence indicates that using an aromatase inhibitor rather than tamoxifen with OFS, reduces the risk of breast cancer recurrence [7]. In postmenopausal patients, aromatase inhibitors (AI) for at least 5 years [7] are recommended. Extended treatment is discussed for patients at intermediate or high risk of recurrence [8,9,10] in both settings.

A consistent direct association between obesity (defined as a BMI value ≥ 30 kg/m^2^) and the risk of developing BC has been observed [11], although this association varies depending on menopause and estrogen receptor (ER) status. Indeed, in pre-menopause the obesity-related increased risk is higher for triple-negative BC (TNBC) and lower for ER-positive BC women, while the opposite occurs post menopause [12,13]. Postmenopausal obese patients not only have a 30% increased risk of developing BC [11] but also a worse prognosis in terms of both DFS and OS [14]. In this regard, a BMI ≥ 40 kg/m^2^ has proven associated to increased mortality [15].

In postmenopausal women, the production of estrogen is mostly ensured in extragonadal sites, mainly in the adipose tissue, where androgens provide the substrates for adipocyte-derived aromatase for estrogen synthesis [16] that exert a direct cancerogenic effect by stimulating the growth of breast tissue through binding to their receptor. An indirect protumoral action is sustained in the pro-estrogenic environment favoring inflammation by molecules produced as a result of their metabolism [17], including IL-6 that further stimulates aromatase expression [18].

In light of all these considerations, it can be hypothesized that the efficacy of adjuvant AI treatment might be impaired by obesity in postmenopausal BC patients. In order to verify this hypothesis we sought to identify a specific cut-off of BMI impacting the risk of recurrence in BC patients with radically resected tumors undergoing systemic adjuvant therapy with AI and verified the AI-specificity of the cut-off by analyzing three other ‘non-AI’ cohorts, namely Tamoxifen-treated HR+ BC, TNBC, and colorectal cancer (CRC). Differences in candidate biomarkers between obese and non-obese patients in the AI cohort were also evaluated.

## 2. Materials and Methods

### 2.1. Patients

**Figure 1:**
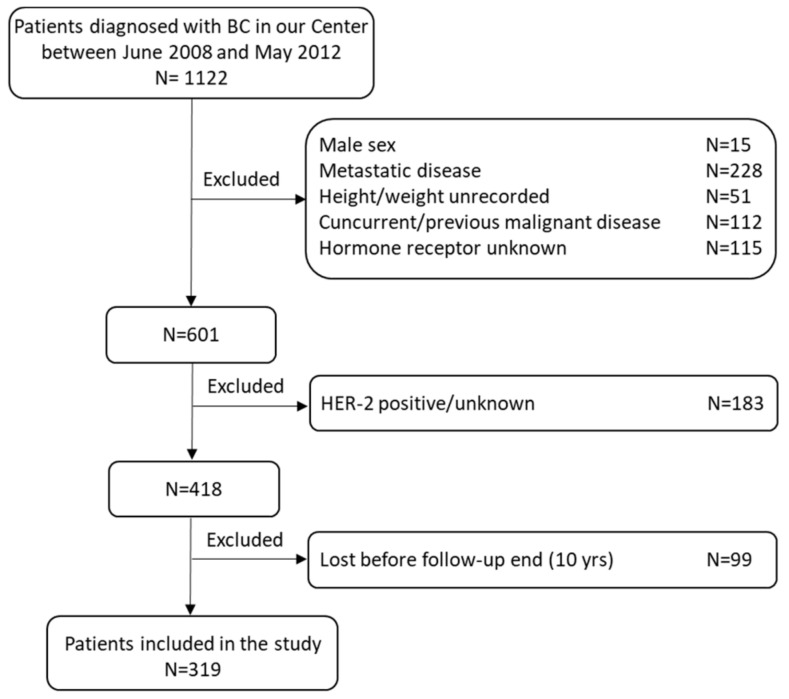
Study flow chart.

A retrospective evaluation of clinical data of 1122 pre- and post-menopausal patients with confirmed histological diagnoses of infiltrating non-metastatic breast cancer who were referred to the Oncology Unit of “Tor Vergata” University Hospital was carried out. The patient cohort consisted of early-stage (I-II) BC patients who had undergone radical upfront BCT, followed by adjuvant radiotherapy treatment and either adjuvant or neoadjuvant chemotherapy, as established by current guidelines. Patients underwent chemotherapy depending on the tumor histotype, the extent of the tumor (size and involvement of loco-regional lymph nodes), estrogen/progesterone receptor (ER/PR) expression, and the cell proliferation index Ki67. HER2-positive and metastatic BC patients were not included in the analysis (Figure 1).

A further cohort of 116 female patients with radically resected colorectal cancer (CRC) undergoing adjuvant chemotherapy was chosen to represent the “Non-breast” control group.

Baseline routinely collected variables and anthropometric parameters to derive BMI [weight (Kg)/height^2^ (m^2^)] were acquired for all patients included in the study. In addition, data on variables routinely collected at the moment hormone therapy or chemotherapy started were collected to analyse differences between patient subgroups.

### 2.2. Statistical Analysis

The maximally selected rank statistics (MSRS), which allow the evaluation of the division points of a continuous variable and provide the classification of the observations in two groups, were used to identify the optimal BMI cut-off for the impact on DFS in the AI-treated BC patients.

A hazard ratio (HR)-smoothed curve analysis using BMI as continuous variable was then performed to assess for non-linear association between BMI and DFS in the AI cohort and visually inspect for the reliability of the cut-off identified with the MSRS analysis.

The impact of the BMI cut-off on the probability of survival was estimated with the method of Kaplan–Meier, with log-rank test and hazard ratio estimation comparing patient subgroups. Impact of the identified BMI cut-off was analysed in all the patient cohorts in order to evaluate their AI-specificity. Furthermore, in the cohort of patients treated with AI, possible differences between the three commonly used AI (Anastrozole, Letrozole and Exemestane) [19], were assessed.

The association between common variables collected at baseline and BMI categories in the AI cohort was analysed by means of the Mann–Whitney U test.

All variables were considered significant for *p* values < 0.05. Data were analysed using the R software (version 4.2.2) and MedCalc.

## 3. Results

A total of 319 female patients with stage I-IIA BC, all treated with radical BCT and RT, followed by adjuvant treatment endocrine therapy, chemotherapy, or both, were included in the study. Neoadjuvant chemotherapy before surgery was allowed. BC was staged according to the TNM classification. Lymph node invasion was negative in 45.4% cases (*n* = 145), while expression of estrogen or progesterone receptors was positive in the majority of patients (*n* = 254; 79.6%). Sixty-five patients with TNBC received only chemotherapy and represented the “hormone-insensitive” group of patients. The median age at the time of the breast cancer diagnosis was 54 years (range 29–85), 54 years (range 29–85) in the “hormone-sensitive” group and 56 (range 40–74) in the “hormone-insensitive” group. According to menopausal status, 223 patients (69.9%) were in post-menopause (80% in TNBC) and 96 (30.1%) in pre-menopause (20% in TNBC). Overweight/obesity was observed in 160 cases (50.2%) in the whole population, in 37 cases (56.8%) in TNBC patients. Patients’ gynecological history was recorded. Forty-four women reported use of either hormone replacement therapy (*n* = 10) or oral contraceptives (*n* = 34). These patients were distributed among the different groups without statistical significance (*p* = 0.96). In all hormone-sensitive BC patients menopause was induced through OFS either by chemotherapy or by LHRH analogues. Patients’ characteristics at the time of diagnosis are summarized in Table 1.

For the “non-breast” control group, a cohort of 116 female patients (mean age 61 years; range 25–81) with radically resected non-metastatic colorectal cancer (CRC) who underwent adjuvant chemotherapy with 5-Fluorouracile alone (18%) or in combination with Oxaliplatin (82%), was evaluated. Obesity/overweight was observed in 44% cases (*n* = 54). According to menopausal status, 31 patients (26.7%) were in pre-menopause and 85 (73.3%) were in post-menopause. The clinical and demographic characteristics of these patients are summarized in Table 2.

All patients were regularly seen at their scheduled follow-up visits or at the occurrence of any clinical suspect of recurrence, and were followed-up for a median period of 71.6 months. The study was performed in accordance with the principles embodied in the Declaration of Helsinki. All patients gave written informed consent for retrospective studies, previously approved by our Institutional Ethics Committees.

### 3.1. BMI Cut-Off Identification

**Figure 2:**
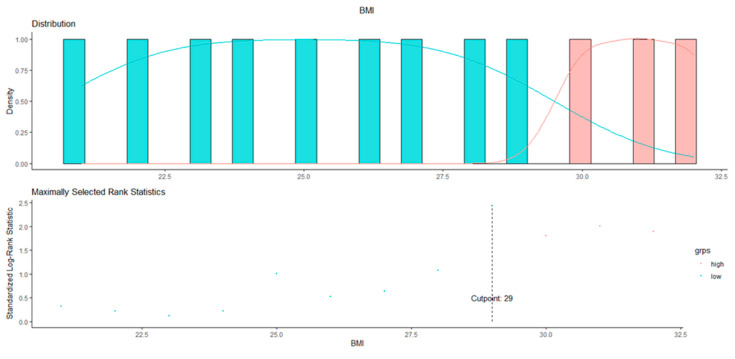
BMI cut-off identification in AI-treated BC patients.

According to the MSRS analysis performed on 172 BC patients receiving adjuvant therapy with aromatase inhibitors, a BMI > 29 was associated with the poorest prognosis in terms of DFS (Figure 2).

**Figure 3:**
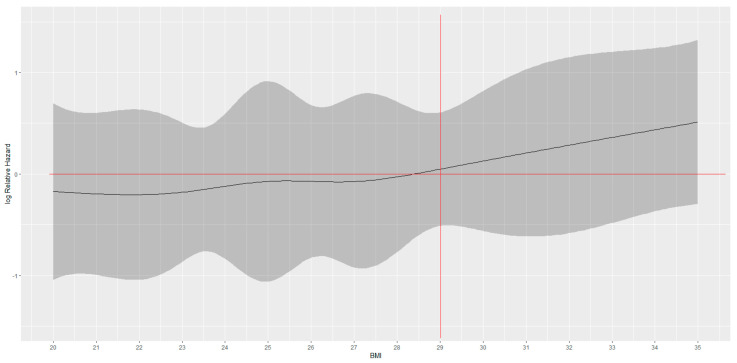
DFS hazard-ratio-smoothed curve for increasing values of BMI. Curve-fitting analysis showed that for BMI cut-off > 29 in AI-treated BC patients DFS was increasingly deteriorating.

Through curve-fitting, which defines the “best fit” model of the relationship between BMI and survival, it was then demonstrated that for BMI values between 20 and 28 kg/m^2^ the HR remains more or less constant. For BMI values > 28 kg/m^2^ and ≤31 kg/m^2^, however, the curve slope begins to change and then tends to rise continuously upwards up to values of BMI = 35 kg/m^2^. This highlights that for high BMI values, patients present an increased risk of local and distant recurrence, which, therefore, determines a worse prognosis than for lower BMI (Figure 3).

### 3.2. Impact of BMI on Survival

**Figure 4:**
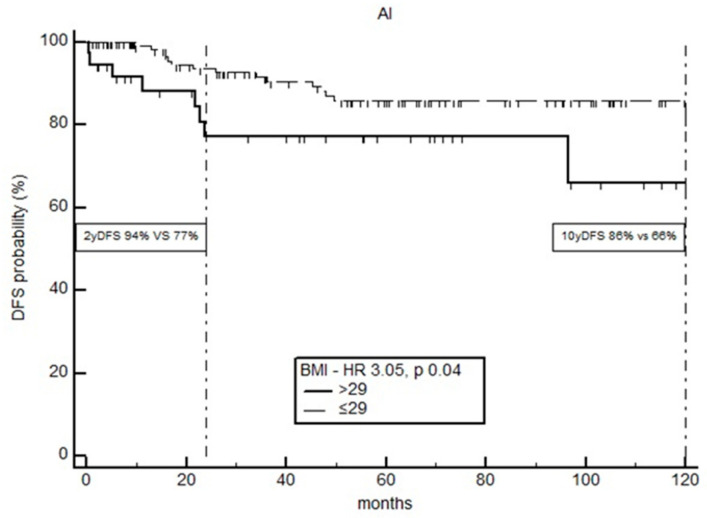
Two- and ten-year disease-free survival of BC patients stratified according to BMI cut-off value.

As regards the impact of BMI on 2-year disease-free survival (2yDFS), from the analysis of the Kaplan–Meier curves emerged that the identified BMI cut-off is specific only for the cohort of postmenopausal BC patients under adjuvant therapy with AI [HR 3.05 (95%CI; 1.05–8.82)] (*p* = 0.04). In fact, for this set of patients, the presence of a BMI ≥ 29 kg/m^2^ causes a 3-fold increased risk of recurrence compared to a BMI < 29 kg/m^2^ (HR, 3.05; 95%CI, 1.05–8.82), with a 2yDFS of 94% vs. 77%, respectively. If the follow-up of patients treated with AI is protracted at 10 years, only 66% of patients with BMI ≥ 29 kg/m^2^ are free from disease (Figure 4).

The effect of BMI on DFS was independent of the AI used, since no significant difference was found between BMI and the specific AI employed (Anastrozole, Letrozole or Exemestane) (data not shown).

**Figure 5:**
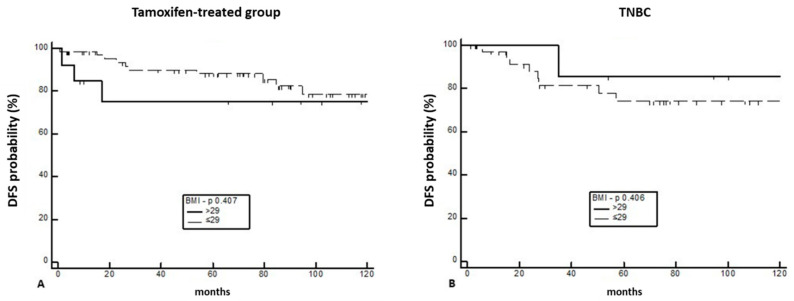
Two-year DFS of BC patients treated with Tamoxifen (**A**) or without endocrine therapy due to TNBC (**B**).

Regarding the 2yDFS of BC patients treated with Tamoxifen [HR 1.90 (95%CI; 0.41–8.80)] (*n* = 82), or not treated with endocrine therapy because of triple-negative BC [HR 0.52 (95%CI; 0.11–2.42)] (*n* = 65) no statistically significant difference was observed when comparing patients with BMI greater or lesser than 29 (*p* = 0.407 and 0.406, respectively) (Figure 5A,B).

**Figure 6:**
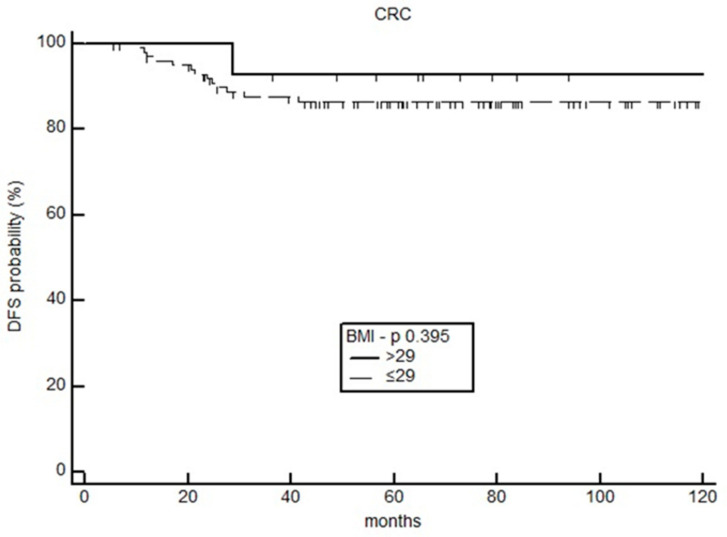
Disease-free survival in CRC patients.

The 2yDFS analysis was also carried out in the cohort of 116 patients with radically resected non-metastatic colorectal cancer (CRC) under 5-Fluorouracil-based regimens. The survival analysis did not demonstrate any significant difference between patients with a BMI ≥ 29 kg/m^2^ and the those with lower BMI values [HR 0.53 (95%CI; 0.12–2.25), *p* = 0.394] (Figure 6).

### 3.3. Biochemical and Histopathological Parameters

We then focused on identifying the biochemical parameters and histopathological characteristics of the BC patient cohort treated with AI (*n* = 172) who had BMI values greater or lesser than 29 kg/m^2^ to generate a hypothesis on possible reasons for the detrimental effect of obesity on the efficacy of adjuvant AI. Among variables commonly registered at baseline prior to the start of the therapy in order to asses hematological, hepatic and renal functions, plus the lipid and mineral balance of AI-treated BC patients, seven parameters resulted statistically different between patients with BMI greater or lesser than 29 kg/m^2^ Table 3.

## 4. Discussion

Breast cancer is the main cancer diagnosed in women and the second most common cause of cancer death worldwide [20,21]. Meta-analyses show an increased risk of developing a hormone-positive BC after menopause, as well as an increase in the risk of recurrence or death and a shorter DFS in obese women, as compared to normal weight ones [22]. Post-menopause, the main substrate for estrogen production is aromatase-mediated androgen conversion in the adipose tissue [16]. For this reason, BC patients with estrogen-receptor-expressing BC, regardless of whether or not they have undergone chemotherapy, are treated with aromatase inhibitors. Two clinical trials, the ATAC study [23] and the ASCSG-6a study [24], evaluated the efficacy of hormone therapy on hormone-sensitive BC. The ATAC study involved postmenopausal women with early ER+ BC and compared the effects of an adjuvant, Anastrozole-based therapy with Tamoxifen-based treatment and demonstrated that BC patients treated with Anastrozole had lower relapse rates than those in the Tamoxifen group. This result was observed for all BMI groups, although the greatest benefit was found in women with a low BMI (<23 kg/m^2^) [23]. On the contrary, the effect of Tamoxifen was comparable for all BMI groups considered. The ASCSG-6a study evaluated the effects of a further 3-year extended treatment with Anastrozole and showed that the addition of Anastrozole in normal weight women halved the risk of relapse but such benefit was lost in overweight or frankly obese women [24].

The relationship between obesity, breast cancer incidence/recurrence and prognosis is complex and may imply both direct mechanisms involving insulin, inflammation and estrogen levels and indirect mechanisms, including inadequate AI dosage, reduced compliance with therapy or insufficient inhibition of excess aromatase present in the adipose tissue of obese subjects [25]. The effectiveness of aromatase inhibitors is related to the amount of enzymes and the degree of estrogen suppression. Therefore, it is conceivable that in obese women there may be an inadequate suppression of aromatase due to the greater amount of adipose tissue present. This concept is supported by studies showing a reduced effect inhibitory of AI in obese subjects undergoing AI therapy [26]. This reduction in efficacy is not observed in subgroups of obese patients receiving Tamoxifen that acts at the level of the ER and, therefore, is not affected by the major aromatization capacity of obese patients.

In obese patients, particularly in post-menopause, also the metabolic syndrome incidence is strongly associated with an increased risk of developing BC. A notable correlation has been found between some conditions that are often found in obese subjects (such as hyperglycemia, low cholesterol levels HDL, hypertriglyceridemia and increase in central adipose tissue) and the configuration of a clinical picture characterized by chronic systemic inflammation, a condition that promotes tumor genesis and growth [27]. There is biological evidence that glucose and other factors related to glucose metabolism, such as insulin and IGF3, contribute to the development of breast tumors, and a strong association between fasting blood glucose and the risk of breast cancer in pre- and post-menopausal women, particularly if obese has been described [28]. In our study, patients with the worst prognosis fell under the classification of metabolic syndrome according to WHO 1999 criteria, as they showed levels of blood glucose> 110 mg/dL and BMI values >30 kg/m^2^. In the population examined in the present study, confirming previous observations [29], the increase in glucose concentration was not associated with liver functions, such as AST and ALT, or other indices of residual liver function, such as bilirubin and γGT. In contrast, patients with BMI > 29 kg/m^2^ had higher fasting blood glucose levels compared to patients with BMI < 29 kg/m^2^ (*p* = 0.004). Increased levels of creatinine, although still within a normal range, resulted in any case statistically significant (*p* = 0.017) in patients with BMI > 29 kg/m^2^. This datum might be consistent with what demonstrated in a prospective cohort study of 1713 participants in the chronic analysis renal insufficiency cohort (CRIC) in which the researchers identified a correlation between impaired renal function and fasting hyperglycemia, recording over time an increased incidence of developing diabetes [30]. In support of the correlation between inflammation and obesity, as well as the importance of an inflammatory microenvironment with regards to carcinogenesis and tumor growth, patients with BMI ≥ 29 kg/m^2^ presented fibrinogen levels (albeit in the normal range) higher than those measured in patients with BMI < 29 kg/m^2^.

Several mechanisms contribute to the correlation between high cholesterol levels and increased growth of breast cancer. Since cholesterol is an important component of the plasma membrane, rapidly dividing cells, such as cancerous ones, require large quantities of this steroid for membrane synthesis: elevated levels of circulating cholesterol could provide the substrate for cell proliferation. Cholesterol is also the precursor to many sex hormones, including progesterone, estrogen, androgen and their derivatives, and it has been speculated that cholesterol could be used by cancer cells to synthesize sex hormones and cause resistance to systemic anti-hormone therapies [31]. In our study, higher cholesterol levels were found in patients with BMI > 29 kg/m^2^ compared to those who had a BMI < 29 kg/m^2^ (*p* = 0.031). Obesity, reduced glucose intolerance, dyslipidemia and elevated blood pressure are closely related not only to increased risk to develop breast cancer, but also to the development of a disease with a more severe prognosis [32,33,34]. The presence of “unfavorable” biochemical characteristics seems also associated to the presence of more biologically aggressive tumors. Accordingly, in our study patients with BMI ≥ 29 kg/m^2^ had higher Ki67 levels, index of cell proliferation and prognostic factor for disease recurrence, higher than those observed in patients with BMI < 29 kg/m^2^ (*p* = 0.01).

A protective role against carcinogenesis in cancer breast cells expressing ER appears to be played by Vit.D, which exerts an important inhibitory action on cell proliferation, inducing cell death and causing cell cycle arrest [35]. In particular, in ER-positive tumors, Vit.D down-regulates ER receptor expression [36,37]. Moreover, Vit.D has shown synergistic effects with anticancer drugs [38]. For these reasons, the addition of Vit.D to AI treatment could be doubly advantageous, both in counteracting the side effects at the bone level that occur during AI administration, and increasing the degree of aromatase suppression at the level of breast cancer cells and surrounding adipose tissue [16,39]. Conversely, Vit.D deficiency is linked to a reduction in DFS and OS in BC patients [40,41]. Several epidemiological studies proved that obese patients have decreased blood levels of vitamin D (Vit.D) [42,43], and that in cancer patients, for every 1 kg/m^2^ increase in BMI there is a decrease in vitamin D of about 1 nmol/L [43,44,45,46,47,48]. A number of factors, including the sequestration of this molecule in adipose tissue [49], can explain the association between the concomitant increase in BMI and decrease in Vit.D. Accordingly, our data show that higher Vit.D levels are observed in patients with better DFS and a BMI < 29 kg/m^2^, compared to obese patients (*p* = 0.0001).

The worst prognosis of patients with BMI > 29 kg/m^2^ appears to be related to an insufficient inhibition of aromatase and, therefore, not simply to a condition of obesity. This datum is confirmed by the observation that the association between BMI and reduced DFS is not found in patients diagnosed with triple-negative breast cancer that, therefore, are not subject to anti-hormone treatment due to a lack of ER/PR expression, and not even in colorectal cancer patients. Our result is in contrast with a recently published observation by Pantelimon et al. demonstrating that patients diagnosed with aggressive tumors subtypes (HER2-positive and TNBC) had a significantly higher BMI than luminal-type BC patients [50]. However, the small number of patients with such conditions (7 HER2-positive and 5 TNBC, respectively, vs. 27 luminal BC patients) included in the cited study [50] does not allow a proper comparison.

The choice to use a 2yDFS interval instead of the more classic 5-year DFS, stems from the observation that obese patients under endocrine treatment have a higher rate of recurrence that increases with increasing BMI, with survival curves that already part after 2 years [34]. Moreover, since TNBC patients have a more unfavorable prognosis at 5 years, the datum would have been masked by the cancer aggressiveness in such population. It has in fact been shown that after adjuvant or neoadjuvant chemotherapy, 5-year overall survival (OS) for TN tumors is 64% (95% CI, 44–79%) compared to 85% (95% CI, 79–90%) observed for the other molecular subtypes, and the 5-year DFS (5yDFS) for TNBC was 57% (95% CI, 37–73%) compared to 72% (95% CI, 64–78%) [51].

The present study has several limitations that have to be taken into account or consideration when interpreting the results. First, it is a single-center study. Second, the sample only represented the white ethnic group. Indeed, several studies underlined the disparity between the weight status by race/ethnicity, with Asian women having different risk profiles compared to whites [52], developing Type 2 diabetes at a lower BMI [53] and have a significant impact on BC risk and survival [54]. Finally, the change in BMI was recorded only throughout the first 5-year follow-up, so we cannot have correlations over a longer DFS.

## 5. Conclusions

In conclusion, in post-menopause (either physiological or treatment-induced) patients with radically resected hormone-sensitive BC, undergoing neoadjuvant or adjuvant chemotherapy and treated with AI, obesity represents a risk factor for early recurrence of the disease, with a significantly reduced DFS, particularly at 2 years. The presence of parameters that configure a condition of metabolic syndrome represent a further disadvantage in obese patients with a worse prognosis.

With a view to developing predictive models of risk of relapse, it could be useful to include BMI, with a cut-off of 29 kg/m^2^, among the criteria to be taken into consideration. Registering the eventual changes in BMI throughout the follow-up might represent an adjunctive tool to refine such risk.

## Figures and Tables

**Table 1:** Non metastatic, HER2-negative BC patients’ clinical and pathological characteristics.

Variable	N (%)
Number of patients	319
Age at diagnosis (mean and range)	53.7 (29.2–85.1)
Menopausal status	
Pre-menopause	96 (30.1)
Post-menopause	223 (69.9)
BMI	
Underweight (<18.5)	8 (2.5)
Normal weight (18.5–24.99)	151 (47.3)
Overweight (25–29.99)	101 (31.7)
Obese (≥30)	59 (18.5)
Histology	
Ductal	273 (85.6)
Lobular	40 (12.5)
Other	6 (1.9)
Grading	
G1	44 (13.8)
G2	134 (42)
G3	141 (44.2)
TNM	
T1N0	77 (24.1)
T2-T3N0	68 (21.3)
N+	174 (54.6)
Molecular subtype	
Luminal A-like	103 (32.3)
Luminal B-like	151 (47.3)
Triple-negative	65 (20.4)
ECOG-PS	
0	315 (98.7)
1	4 (1.3)
Chemotherapy	
Neoadjuvant	101 (31.6)
Adjuvant	218 (68.4)
Chemotherapy Scheme	
Antracycline	88 (27.6)
Antracycline + Taxanes	198 (62)
Taxanes	33 (10.4)
Endocrine therapy	
Aromatase inhibitors	172 (67.7)
Tamoxifene	82 (32.3)
Type of AI	
Anastrozole	122 (71)
Letrozole	32 (18.6)
Exemestane	18 (10.4)

AI: Aromatase inhibitors; ECOG-PS: Eastern Cooperative Oncology Group—performance status.

**Table 2:** Non metastatic, colorectal cancer patients’ clinical and pathological characteristics.

Variable	N (%)
Number of patients	116
Age at diagnosis (mean and range)	61 (34.7–80.6)
Menopausal status	
Pre-menopause	31 (26.7)
Post-menopause	85 (73.3)
BMI	
Underweight (<18.5)	6 (5)
Normal weight (18.5–24.99)	59 (51.0)
Overweight (25–29.99)	37 (32.0)
Obese (>30)	14 (12.0)
Chemotherapy Scheme	
5-Fluorouracil	21 (18.0)
5-Fluorouracil + Oxaliplatin	95 (82)

**Table 3:** Biochemical parameters of postmenopausal BC patients stratified according to BMI prior to AI treatment start.

	BMI < 29	BMI > 29	
Variable	Mean Value	Mean Value	*p*
Fibrinogen (mg/dL)	292	335	<0.0001
Glucose (mg/dL)	95	119	0.004
ALT (UI/L)	18	17	0.67
AST (UI/L)	18	18	0.89
γGT (UI/L)	21	24	0.92
Bilirubin (mg/dL)	0.44	0.40	0.47
Urea (mg/dL)	30	34	0.04
Creatinine (mg/dL)	0.7	0.8	0.01
Total Cholesterol (mg/dL)	194	209	0.03
Triglycerides (mg/dL)	125	117	0.68
Vitamin D (UI/L)	32	26	0.0001
Ki-67 expression (%)	20%	29%	0.01

## Data Availability

Not applicable.

## References

[1] Perou C.M., Sørlie T., Eisen M.B., van de Rijn M., Jeffrey S.S., Rees C.A., Pollack J.R., Ross D.T., Johnsen H., Akslen L.A. (2000). Molecular portraits of human breast tumours. Nature.

[2] Yersal O., Barutca S. (2014). Biological subtypes of breast cancer: Prognostic and therapeutic implications. World J. Clin. Oncol..

[3] Ratosa I., Plavc G., Pislar N., Zagar T., Perhavec A., Franco P. (2021). Improved Survival after Breast-Conserving Therapy Compared with Mastectomy in Stage I-IIA Breast Cancer. Cancers.

[4] Burstein H.J., Curigliano G., Thürlimann B., Weber W.P., Poortmans P., Regan M.M., Senn H.J., Winer E.P., Gnant M., Panelists of the St Gallen Consensus Conference (2021). Customizing local and systemic therapies for women with early breast cancer: The St. Gallen International Consensus Guidelines for treatment of early breast cancer 2021. Ann Oncol..

[5] Smith I.E., Dowsett M. (2003). Aromatase inhibitors in breast cancer. N. Engl. J. Med..

[6] Conte B., Poggio F., Del Mastro L. (2017). Luteininzing hormone releasing hormones analogs in combination with tamoxifen for the adjuvant treatment of premenopausal women with hormone receptor positive breast cancer. Expert Opin. Pharmacother..

[7] Early Breast Cancer Trialists’ Collaborative Group (EBCTCG) (2022). Aromatase inhibitors versus tamoxifen in premenopausal women with oestrogen receptor-positive early-stage breast cancer treated with ovarian suppression: A patient-level meta-analysis of 7030 women from four randomised trials. Lancet.

[8] Sella T., Ruddy K.J., Carey L.A., Partridge A.H. (2021). Optimal Endocrine Therapy in Premenopausal Women: A Pragmatic Approach to Unanswered Questions. JCO Oncol. Pract..

[9] Del Mastro L., Mansutti M., Bisagni G., Ponzone R., Durando A., Amaducci L., Campadelli E., Cognetti F., Frassoldati A., Michelotti A. (2021). Extended therapy with letrozole as adjuvant treatment of postmenopausal patients with early-stage breast cancer: A multicentre, open-label, randomised, phase 3 trial. Lancet Oncol..

[10] Gnant M., Fitzal F., Rinnerthaler G., Steger G.G., Greil-Ressler S., Balic M., Heck D., Jakesz R., Thaler J., Egle D. (2021). Duration of adjuvant aromatase-inhibitor therapy in postmenopausal breast cancer. N. Engl. J. Med..

[11] Reeves G.K., Pirie K., Beral V., Green J., Spencer E., Bull D., Million Women Study Collaboration (2007). Cancer incidence and mortality in relation to body mass index in the Million Women Study: Cohort study. BMJ.

[12] Smith S.G., Sestak I., Morris M.A., Harvie M., Howell A., Forbes J., Cuzick J. (2021). The impact of body mass index on breast cancer incidence among women at increased risk: An observational study from the International Breast Intervention Studies. Breast Cancer Res. Treat..

[13] Picon-Ruiz M., Morata-Tarifa C., Valle-Goffin J.J., Friedman E.R., Slingerland J.M. (2017). Obesity and adverse breast cancer risk and outcome: Mechanistic insights and strategies for intervention. CA A. Cancer J. Clin..

[14] Sparano J.A., Wang M., Zhao F., Stearns V., Martino S., Ligibel J.A., Perez E.A., Saphner T., Wolff A.C., Sledge G.W. (2012). Obesity at diagnosis is associated with inferior outcomes in hormone receptor-positive operable breast cancer. Cancer.

[15] Calle E.E., Rodriguez C., Walker-Thurmond K., Thun M.J. (2003). Overweight, Obesity, and Mortality from Cancer in a Prospectively Studied Cohort of U.S. Adults. N. Engl. J. Med..

[16] Simpson E.R., Clyne C., Rubin G., Boon W.C., Robertson K., Britt K., Speed C., Jones M. (2002). Aromatase—A Brief Overview. Annu. Rev. Physiol..

[17] Yager J.D., Davidson N.E. (2006). Estrogen Carcinogenesis in Breast Cancer. N. Engl. J. Med..

[18] Simpson E.R., Brown K.A. (2013). Minireview: Obesity and breast cancer: A tale of inflammation and dysregulated metabolism. Mol. Endocrinol..

[19] De Placido S., Gallo C., De Laurentiis M., Bisagni G., Arpino G., Sarobba M.G., Riccardi F., Russo A., Del Mastro L., Cogoni A.A. (2018). Adjuvant anastrozole versus exemestane versus letrozole, upfront or after 2 years of tamoxifen, in endocrine-sensitive breast cancer (FATA-GIM3): A randomised, phase 3 trial. Lancet Oncol..

[20] Sung H., Ferlay J., Siegel R.L., Laversanne M., Soerjomataram I., Jemal A., Bray F. (2021). Global Cancer Statistics 2020: GLOBOCAN estimates of Incidence and mortality worldwide for 36 cancers in 185 countries. CA A. Cancer J. Clin..

[21] Bray F., Ferlay J., Soerjomataram I., Siegel R.L., Torre L.A., Jemal A. (2018). Global cancer statistics 2018: GLOBOCAN estimates of incidence and mortality worldwide for 36 cancers in 185 countries. CA A Cancer J. Clin..

[22] Protani M., Coory M., Martin J.H. (2010). Effect of obesity on survival of women with breast cancer: Systematic review and meta-analysis. Breast Cancer Res. Treat..

[23] Gnant M., Pfeiler G., Stoger H., Mlineritsch B., Fitzal F., Balic M., Kwasny W., Seifert M., Stierer M., Dubsky P. (2013). The predictive impact of body mass index on the efficacy of extended adjuvant endocrine treatment with anastrozole in postmenopausal patients with breast cancer: An analysis of the randomised ABCSG-6a trial. Br. J. Cancer.

[24] Sestak I., Distler W., Forbes J.F., Dowsett M., Howell A., Cuzick J. (2010). Effect of body mass index on recurrences in tamoxifen and anastrozole treated women: An exploratory analysis from the ATAC trial. J. Clin. Oncol..

[25] Niraula S., Ocana A., Ennis M., Goodwin P.J. (2012). Body size and breast cancer prognosis in relation to hormone receptor and menopausal status: A meta-analysis. Breast Cancer Res. Treat..

[26] Geisler J., Haynes B., Anker G., Dowsett M., Lønning P.E. (2002). Influence of letrozole and anastrozole on total body aromatization and plasma estrogen levels in postmenopausal breast cancer patients evaluated in a randomized.; cross-over study. J. Clin. Oncol..

[27] Iyengar N.M., Gucalp A., Dannenberg A.J., Hudis C.A. (2016). Obesity and Cancer Mechanisms: Tumor Microenvironment and Inflammation. J. Clin. Oncol..

[28] Muti P., Quattrin T., Grant B.J., Krogh V., Micheli A., Schünemann H.J., Ram M., Freudenheim J.L., Sieri S., Trevisan M. (2002). Fasting glucose is a risk factor for breast cancer: A prospective study. Cancer Epidemiol. Biomarkers Prev..

[29] Takeda Y., Fujita Y., Bessho R., Sato M., Abe T., Yanagimachi T., Sakagami H., Abiko A., Takiyama Y., Ota T. (2019). Increment of plasma glucose by exogenous glucagon is associated with present and future renal function in type 2 diabetes: A retrospective study from glucagon stimulation test. BMC Endocr. Disord..

[30] Jepson C., Hsu J.Y., Fischer M.J., Kusek J.W., Lash J.P., Ricardo A.C., Schelling J.R., Feldman H.I. (2019). Chronic Renal Insufficiency Cohort (CRIC) Study Investigators. Incident Type 2 Diabetes Among Individuals With CKD: Findings From the Chronic Renal Insufficiency Cohort (CRIC) Study. Am. J. Kidney Dis..

[31] Desai P., Lehman A., Chlebowski R.T., Kwan M.L., Arun M., Manson J.E., Lavasani S., Wasswertheil-Smoller S., Sarto G.E., LeBoff M. (2015). Statins and breast cancer stage and mortality in the Women’s Health Initiative. Cancer Causes Control..

[32] Kabat G.C., Kim M.Y., Lee J.S., Ho G.Y., Going S.B., Beebe-Dimmer J., Manson J.E., Chlebowski R.T., Rohan T.E. (2017). Metabolic obesity phenotypes and risk of breast cancer in postmenopausal women. Cancer Epidemiol. Biomark. Prev..

[33] Ferroni P., Riondino S., Buonomo O., Palmirotta R., Guadagni F., Roselli M. (2015). Type 2 Diabetes and Breast Cancer: The Interplay between Impaired Glucose Metabolism and Oxidant Stress. Oxid. Med. Cell. Longev..

[34] Dibaba D.T., Ogunsina K., Braithwaite D., Akinyemiju T. (2019). Metabolic syndrome and risk of breast cancer mortality by menopause, obesity, and subtype. Breast Cancer Res. Treat..

[35] Zheng W., Cao L., Ouyang L., Zhang Q., Duan B., Zhou W., Chen S., Peng W., Xie Y., Fan Q. (2019). Anticancer activity of 1,25-(OH)2D3 against human breast cancer cell lines by targeting Ras/MEK/ERK pathway. Onco. Targets Ther..

[36] Swami S., Krishnan A.V., Feldman D. (2000). 1α,25-Dihydroxyvitamin D3 Down-Regulates Estrogen Receptor Abundance and Suppresses Estrogen Actions in MCF-7 Human Breast Cancer Cells. Clin. Cancer Res..

[37] Swami S., Krishnan A.V., Wang J.Y., Jensen K., Peng L., Albertelli M.A., Feldman D. (2011). Inhibitory Effects of Calcitriol on the Growth of MCF-7 Breast Cancer Xenografts in Nude Mice: Selective Modulation of Aromatase Expression in vivo. Horm. Canc..

[38] Ma Y., Trump D.L., Johnson C.S. (2010). Vitamin D in combination cancer treatment. J. Cancer.

[39] Krishnan A.V., Swami S., Feldman D. (2012). The potential therapeutic benefits of vitamin D in the treatment of estrogen receptor positive breast cancer. Steroids.

[40] Morton M.L., Thompson C.L. (2013). Decreasing 25-hydroxy-vitamin D levels account for portion of the effect of increasing body mass index on breast cancer mortality. Mol. Nutr. Food Res..

[41] Vrieling A., Hein R., Abbas S., Schneeweiss A., Flesch-Janys D., Chang-Claude J. (2011). Serum 25- hydroxyvitamin D and postmenopausal breast cancer survival: A prospective patient cohort study. Breast Cancer Res..

[42] De Pergola G., Martino T., Zupo R., Caccavo D., Pecorella C., Paradiso S., Silvestris F., Triggiani V. (2019). 25 Hydroxyvitamin D Levels are Negatively and Independently Associated with Fat Mass in a Cohort of Healthy Overweight and Obese Subjects. Endocr. Metab. Immune Disord. Drug Targets.

[43] Pereira-Santos M., Costa P.R.F., Assis A.M.O., Santos C.A.S.T., Santos D.B. (2015). Obesity and vitamin D deficiency: A systematic review and meta-analysis. Obes. Rev..

[44] Vashi P.G., Lammersfeld C.A., Braun D.P., Gupta D. (2011). Serum 25-hydroxyvitamin D is inversely associated with body mass index in cancer. Nutr. J..

[45] Lagunova Z., Porojnicu A.C., Grant W.B., Bruland O., Moan J.E. (2010). Obesity and increased risk of cancer: Does decrease of serum 25-hydroxyvitamin D level with increasing body mass index explain some of the association?. Mol. Nutr. Food Res..

[46] McGill A.T., Stewart J.M., Lithander F.E., Strik C.M., Poppitt S.D. (2008). Relationships of low serum vitamin D3 with anthropometry and markers of the metabolic syndrome and diabetes in overweight and obesity. Nutr. J..

[47] Rodriguez-Rodriguez E., Navia B., Lopez-Sobaler A.M., Ortega R.M. (2009). Vitamin D in overweight/obese women and its relationship with dietetic and anthropometric variables. Obesity.

[48] Stein E.M., Strain G., Sinha N., Ortiz D., Pomp A., Dakin G., McMahon D.J., Bockman R., Silverberg S.J. (2009). Vitamin D insufficiency prior to bariatric surgery: Risk factors and a pilot treatment study. Clin. Endocrinol..

[49] Earthman C.P., Beckman L.M., Masodkar K., Sibley S.D. (2012). The link between obesity and low circulating 25-hydroxyvitamin D concentrations: Considerations and implications. Int. J. Obes..

[50] Pantelimon I., Gales L.N., Anghel R.M., Gruia M.I., Nita I., Matei C.V., Bodea D., Stancu A.M., Pirvu E., Radu M.C. (2022). Aspects Regarding the Influence of Obesity on the Molecular Characteristics of Breast Tumors. Cureus.

[51] Saleh R.R., Nadler M.B., Desnoyers A., Meti N., Fazelzad R., Amir E. (2021). Platinum-based chemotherapy in early-stage triple negative breast cancer: A meta-analysis. Cancer Treat. Rev..

[52] Gong S., Wang K., Li Y., Zhou Z., Alamian A. (2021). Ethnic group differences in obesity in Asian Americans in California, 2013–2014. BMC Public Health.

[53] Vicks W.S., Lo J.C., Guo L., Rana J.S., Zhang S., Ramalingam N.D., Gordon N.P. (2022). Prevalence of prediabetes and diabetes vary by ethnicity among U.S. Asian adults at healthy weight, overweight, and obesity ranges: An electronic health record study. BMC Public Health.

[54] Bandera E.V., Maskarinec G., Romieu I., John E.M. (2015). Racial and ethnic disparities in the impact of obesity on breast cancer risk and survival: A global perspective. Adv. Nutr..

